# A facile synthesis of Au-nanoparticles decorated PbI_2_ single crystalline nanosheets for optoelectronic device applications

**DOI:** 10.1038/s41598-018-32038-5

**Published:** 2018-09-14

**Authors:** Mohd. Shkir, I. S. Yahia, V. Ganesh, Y. Bitla, I. M. Ashraf, Ajeet Kaushik, S. AlFaify

**Affiliations:** 1 Advanced Functional Materials and Optoelectronics Laboratory (AFMOL), Department of Physics, College of Science, King Khalid University, Abha, 61413, P.O. Box, 9004 Saudi Arabia; 2 Research Center for Advanced Materials Science (RCAMS), King Khalid University, Abha, 61413, P.O. Box, 9004 Saudi Arabia; 3 Department of Physics, Indian Institute of Science, Bangalore, 560012 India; 4 Centre of Personalized Nanomedicine, Institute of Neuroimmune Pharmacology, Department of Immunology and Nano-Medicine, Herbert Wertheim College of Medicine, Florida International University, Miami, FL 33199 USA; 5 Department of Physics, Faculty of Science, Aswan University, Aswan, 81511 Egypt

## Abstract

This research communication presents a rapid and facile microwave-assisted synthesis of single crystalline nanosheets (SCNSs) of hexagonal lead iodide **(**PbI_2_) decorated with Au nanoparticles, a potential optoelectronics material. Homogeneous low dimensional AuNP decoration in PbI_2_ resulted in a new absorption band at ~604 nm and a shift in band gap from 3.23 to 3.00 eV. The significant enhancement of photoluminescent (PL) intensity observed in the AuNP-PbI_2_ SCNSs is attributed to the coupling of the localized surface plasmon resonanzce of AuNP leading to improved excitation and emission rates of PbI_2_-SCNSs in the region of the localized electromagnetic field. The Au-PbI_2_ SCNSs display a compelling increment in photoconductivity, and its fabricated photodetector showed a stable and switchable photo-response. Due to ease of synthesis and enhanced photoconductivity along with appealing PL features, Au-PbI_2_ SCNS has the potential to be used as a material of choice when fabricating an optoelectronic devices of high performance.

## Introduction

In the current materials research scenario, the atomically thin nanostructures (i.e., nanosheets) of two (2D) or (3D) dimensional materials with a layered structure such as molybdenum di-sulfide, graphene, boron nitride, transition metal dichalcogenide and nano-semiconductors^1–11^ gained a lot of attention due to their promising potentials in industrial-technological applications. Nanoscale lead iodide (PbI_2_), a layered p-type nano semiconductor with a wide band gap of 2.27 eV, is one such important nanostructured material that has been extensively utilized in series of applications including active matrix flat panel imagers, mammography energy range detection, photoconductors, photo-detectors, photovoltaic, sensors, biological labeling and diagnostics, LEDs and PC solar cells, etc.^12–16^. Layered halide PbI_2_ exhibits a large number of polytypes^17^, with PbI_2_ consists of I-Pb-I repeating units stacked along c-axis and the PbI_2_ interlayers that are connected by van der Waals forces. Due to the robust Pb lone-pair s orbital and I p orbital anti-bonding connection, PbI_2_ exhibits ultralow conductivity and high light conversion capability^18^. Recently, Zhang *et al*. reported the growth of ultrathin single crystals of the PbI_2_ materials and studied its photodetector characteristics^19^. Wei *et al*. reported its single crystal growth and studied its visible-light photo-detector application^20^. PbI_2_ nanostructures have been synthesized using various methodologies^21,22^. However, the synthesis of PbI_2_ doped nanosheets using hydrothermal and microwave methods are newly explored^23–25^. In spite of appreciated opto-electronic properties of PbI_2_, there is a significant space to improve its optoelectronic features via nanostructuring and doping with plasmonic nanoparticles. Keeping that in view, efforts to fabricate PbI_2_ nanosheets with the Au dopant as an active and standard plasmonic nanoparticle are urgently required. Nevertheless, accurate nanoengineering of a particularly well-defined morphology of nanostructure is in principle a challenging task and rarely reported for many of the known nanomaterials. The noble metal nanocrystals/nanoparticles like: Gold (Au), Platinum (Pt), and Silver (Ag) are of a captivating class of materials of inimitable chemical and physical possessions that attain countless claims in the field of biosensing, photonics, catalysis etc.^1,26–30^. Au nanoparticles doping of various synthesized key materials is reported in the literature with remarkable modification in the doped materials properties^1,31^. Hence, it can be predicted that the Au noble metal nanoparticles (NPs) may anchor on the PbI_2_ single crystalline nanosheets (SCNSs) and it could possibly extend its functions as an innovative photodetector, photoconductor, optoelectronic and also as a catalytic nanomaterial, which has not been reported on Au doped PbI_2_ so far, however, reported for many other materials previously^22,28,32–35^.

Hence, herein, we report a rapid and facile microwave-assisted synthesis of Au nanoparticles (AuNPs) decorated PbI_2_ SCNSs with uniform hexagonal morphology. The synthesized nanosheets were characterized by the state of the art techniques. The results provide strong evidence of the potential applications of the fabricated AuNPs decorated PbI_2_ SCNSs and may prove its usefulness in optical and visible-light photodetector device applications and also open a new field of investigation on the titled material for future utilizations.

## Materials and Methods

**Figure 1:**
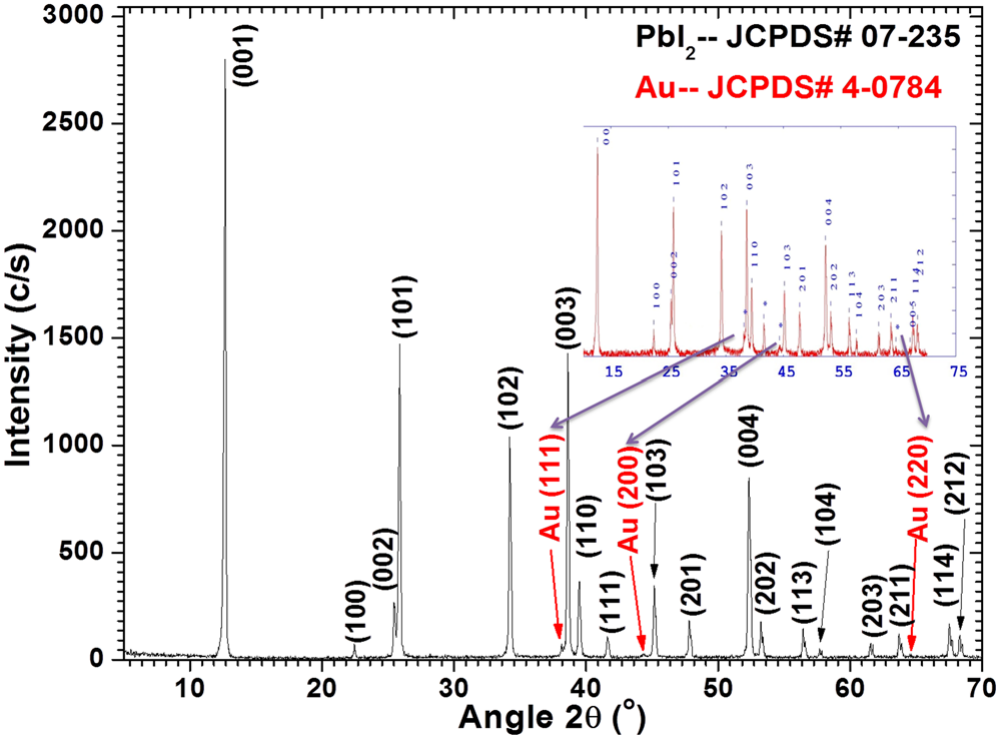
Indexed XRD pattern for Au doped PbI_2_ nanosheets.

Lead acetate [Pb(C_2_H_3_O_2_)_2_], sodium iodide (NaI), Cetyl Trimethyl Ammonium Bromide (CTAB), Gold (III) chloride [AuCl_3_] are taken from Alfa Aesar and Sigma Aldrich Pvt. Ltd. In the first step, 0.5M Pb(C_2_H_3_O_2_)_2_ was taken in 50 ml distilled water in well gutted cylindrical flask and continuously stirred (700 rpm) at constant temperature ~70 °C until a transparent and homogeneous solution was achieved and named as solution A. A stock solution was prepared for 10 g CTAB as surfactant in 1 liter DD water and 50 ml of it was added to solution A, as surfactant played a significant character in preparation of exact shape nanostructured materials^23,36,37^. For Au doping, 1 wt% of Gold (III) chloride was added to the solution A. Next, NaI solution obtained by dissolving 1M NaI in 50 ml water was slowly added, and the formation of a deep yellow precipitate was seen. The yellow precipitate confirms the formation of lead iodide (PbI_2_). For microwave-assisted synthesis, the prepared solution was poured into a cylindrical glass vessel and kept inside a domestic microwave system only for 15 minutes at 700 W, and natural cooling was done. The used domestic microwave system was indigenously modified as shown in Fig. 1 of our previous report^25^. The final product was washed many times with distilled water during the filtration and finally dried at 100 °C in an oven for 24 h.

The investigation of the crystalline structure of the synthesized nanosheets is carried out on Shimadzu X-600 Japan powder X-ray diffractometer (CuK_α_ radiation). DXR FT-RAMAN spectrometer was used to measure the spectra in 600–60 cm^−1^ wavenumber range using 532 nm laser excitation source with only 0.2 mW power. SEM (equipped with EDXS) and TEM is applied on a JSM 6360 and JEOL-EM- 2100 -HR TEM, LA, Japan system to analyze shape, size, spatial spreading of Au NPs and Au doping composition. UV-Visible spectra were documented on a JASCO V-570 UV-Vis-NIR spectrophotometer in 190 to 1000 nm wavelength region. Lumina fluorescence spectrometer was applied to measure photoluminescence (PL) in 350–630 nm range. To study dielectric and alternating current conductivity properties, the capacitance, impedance, and loss tangent measurements were carried out on a KEITHLEY 4200-SCS over a wide frequency range at 300 K. For electrical measurements; the samples were simply designed from prepared doped and undoped PbI_2_ SCNSs as given in scheme I (see Supplementary Data). For photoconductivity measurements, the sample was fixed on the sample holder for optical cryostat (Oxford optistat DN_2_). The inner and outer chambers of the cryostat were evacuated to about 10^−4^ Torr using Edward vacuum system. The desired temperature was achieved using the Oxford temperature controller of model ITC601. The programmable digital electrometer (Keithley 6517b electrometer) used as DC power supply and an ammeter measured the very low current. A tungsten lamp, used for illuminating the sample, was connected to a variac to obtain the desired light intensity. A focusing system consisting of two convex lenses was adjusted between the light source and the sample for obtaining a full and homogeneous illumination on the sample. The experimental arrangement used for the steady-state and transient measurements is shown in Scheme II (see Supplementary Data).

## Results and Discussion

### X-ray diffraction analysis

XRD examination was performed to inspect the excellence, size, and positioning of synthesized Au doped PbI_2_ nanosheets. Figure 1 illustrated a measured and indexed XRD pattern of Au doped PbI_2_ nanosheets. High intensity and sharpness of diffraction peaks confirm good crystallinity of nanosheets and no extra peak due to impurity was seen. The peaks present in the XRD pattern were in agreement with those reported in JCPDS (No. 07–235) for PbI_2_ and (No. 4–0784) for Au. The XRD pattern display the reflection peaks at 12.674° (001), 22.511° (100), 25.506° (002), 25.915° (101), 34.275° (102), 38.674° (003), 39.519° (110), 41.665° (111), 45.209° (103), 52.399° (004), 53.290° (202), 56.487° (113), 57.726° (104), 61.580° (203), 63.751° (211), 67.569° (114), 68.32° (212) that are corresponding to hexagonal phase of PbI_2_ nanostructure with 2H Polytypes^23,38^. Fascinatingly, three additional comparatively low-intensity peaks situated at 38.14°, 44.42°, and 64.6° are allocated to (111), (200) and (220) planes, respectively and are ascribed to FCC structure of Au^39,40^. The intensity of Au diffraction peaks are less compare to PbI_2_ and may improved by annealing. The highest intensity was observed along (001) plane which indicates the growth of single crystalline nanosheets along this plane. The estimated lattice parameters were found to be a = b = 4.5593 Å, c = 6.9872 Å and V = 125.783 Å^3^, which are in agreement with standard PbI_2_ [JCPDS No. 7–0235].

The average values of grain size (L) and strain (ε) were calculated using well-known relations^23^ and are found to be 26.760 nm and 1.38 × 10^−3^, respectively. The calculated value of V is enlarged due to doping compared to pure PbI_2_ nanostructures as reported previously^23,24^. This can be explicated by Vegard’s law, according to this law when doping incorporation takes place on the substitutional or interstitial position in any material the lattice parameters are found to shrivel or inflate, respectively. Due to the smaller ionic radii of Au^3+^ (99 pm)^41^ compared to Pb^2+^ (119 pm), Au gains more possibility to go into the interstitial sites of PbI_2_. The enlarged value of V for Au doped PbI_2_ sample confirms the incorporation of Au in PbI_2_ matrix.

### Vibrational spectroscopy analysis

**Figure 2:**
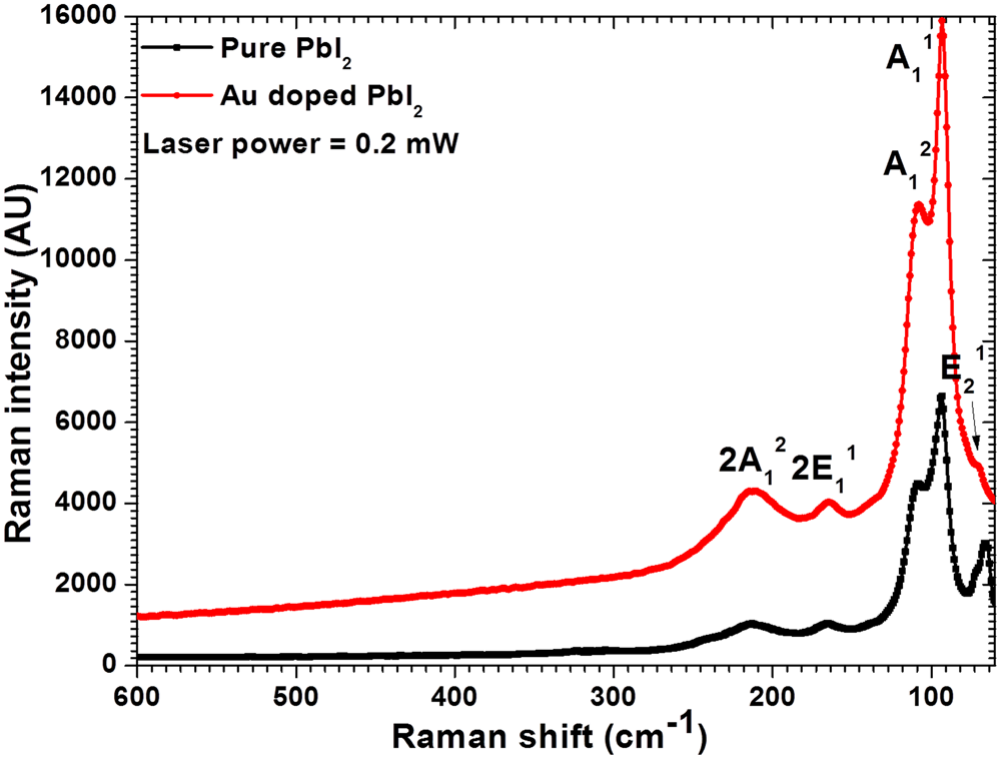
Raman spectra for pure and Au doped PbI_2_ NRs and NSs.

The Raman modes of pure PbI_2_ and Au doped PbI_2_ depicted in Fig. 2 are consistent with 2H polytypes of PbI_2_ predominantly^42,43^. The Raman modes are observed at ~72.5, 94.3, 109.5, 165.77, and 214.9 cm^−1^ for pure PbI_2_ and at ~70.1, 92.8, 107.8, 164.2, 212.8 cm^−1^ for Au doped PbI_2_. The absence of any extra Raman peaks and enhanced intensity confirms the virtuous quality of the prepared product. Raman intensity is found to be remarkably enhanced by Au doping in PbI_2_. Moreover, the full-width half maxima (FWHM) of the highest intensity peak (~93 ± 1 cm^−1^) was calculated for the pure and doped PbI_2_ and found to be ~37.19 and 10.76 cm^−1^, respectively. Furthermore, the under peak areas of these peaks were also evaluated and found to be 35713 and 38555 for the pure and doped product. This clearly indicates that prepared products possess better crystallinity. Enhancement in intensity of lines specifies that nanosheets are of good crystallinity. Moreover, small shifting in Raman modes of nanosheets (Au doped) compares to nanorods (pure) was also observed. As it is known that 2H-PbI_2_ has only 3 atoms in one unit cell and hence have 9 degrees of vibrational motions. Predominantly, 2H-PbI_2_ is expected to have only two bands due to the symmetric stretching A_1g_ band at 97 cm^−1^ and, doubly degenerated E_g_ band at 74 cm^−1^ owing to the shearing motion of two I layers^21^. These bands in the currently synthesized nanostructures are situated at ~92 ± 3 and 72 ± 2 cm^−1^, which are slightly shifted due to nano size structures and also due to more relaxed binding. Redshift and expansion of Raman lines authenticate relaxed nanostructure formation which may be owed to relaxed q- vector. More explanation can be found elsewhere about shifting and expansions in Raman lines^44–46^. It may be mentioned here that no extra peak in the Raman spectrum was observed due to Au doping in PbI_2_. Such results were also reported for Au decorated MoS_2_ as well as other doped materials^1,47^.

### Elemental composition and morphological analyses

**Figure 3:**
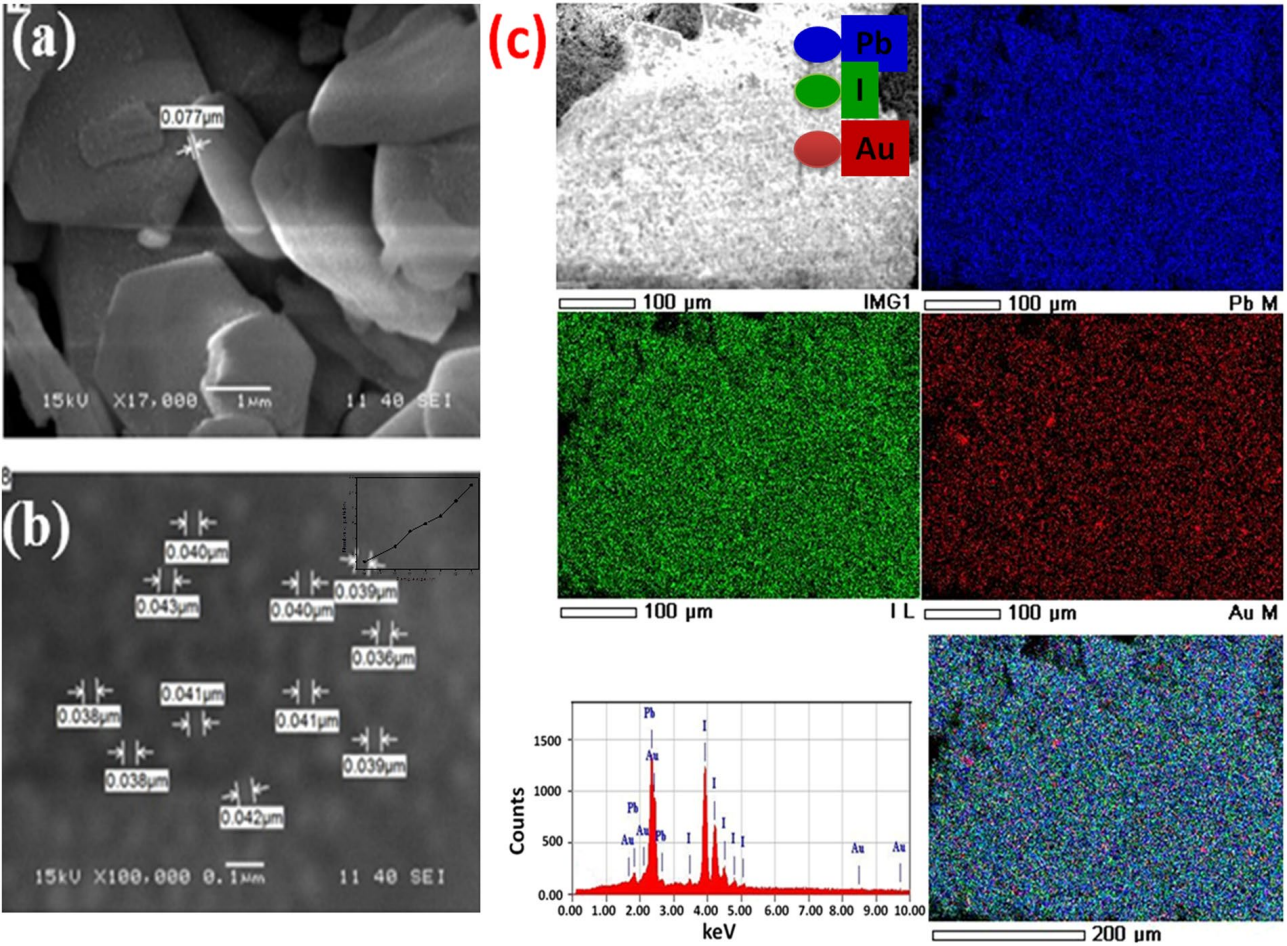
(**a,b**) SEM micrographs and (**c**) SEM mapping of elemental distribution and EDX spectrum of Au doped PbI_2_ SCNSs.

The elemental composition and distribution of Au-PbI_2_ were studied by EDX and SEM mapping analysis. The recorded SEM micrograph of Au-PbI_2_ nanosheets is depicted in Fig. 3(a) which exhibit hexagonal shape. These single crystalline nanosheets have thickness <80 nm and their sizes are in µm range. A close observation of a typical nanosheet in Fig. 3(b) reveals an unvarying spreading of Au nanoparticles on PbI_2_. The average size of Au nanoparticles is less than 40 nm (Histogram is shown in the inset of Fig. 3(b). There is a possibility of error in size as we have measured it manually using SMile View software. Energy dispersive X-ray (EDX) spectroscopy mapping over a representative Au-PbI_2_ nanosheet in Fig. 3(c) shows a homogeneous distribution of the corresponding Pb, I and Au elements. The EDX spectrum of nanosheet is shown in the left bottom panel. The morphologies of pure and doped PbI_2_ nanostructures are reported to show nanoparticles, nanocrystals, nanobelts, nanorods, nanosheets, etc. synthesized by different techniques^23,24,38,48–50^. However, in the present case, the morphology of Au-PbI_2_ still retains a hexagonal nanosheet structure with uniform Au nanoparticles distribution. These Au doped PbI_2_ single crystalline nanosheets could offer incomparable optoelectronic properties and can be used in diverse device applications^10^.

**Figure 4:**
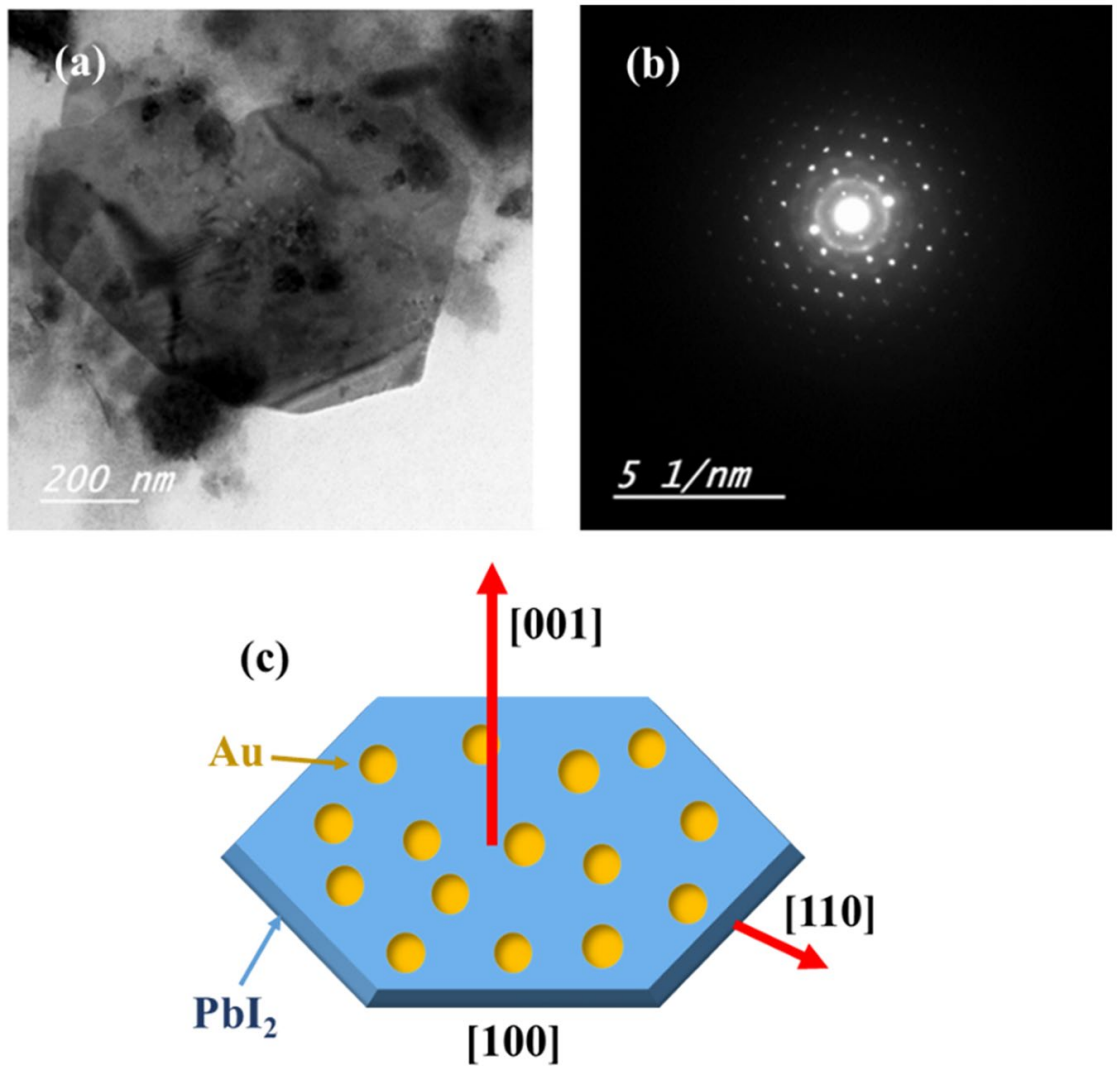
Images of (**a**) HRTEM, (**b**) SAED and (**c**) schematic of Au-PbI_2_ SCNSs.

To get further insight into the microstructure of Au-PbI_2_, high-resolution transmission electron microscopy (HRTEM) study was done. Figure 4(a) shows the HRTEM micrograph of Au-PbI_2_ nanosheet. The selected area electron diffraction (SAED) pattern in Fig. 4(b) shows the dotted pattern of single crystalline PbI_2_ and a polycrystalline ring of Au nanoparticles. Clear diffraction spots of the SAED pattern are consistent with high quality and hexagonal structured single crystalline PbI_2_ nanosheet. Figure 4(c) shows the schematic of the Au-PbI_2_ nanosheet structure.

### Optical studies

**Figure 5:**
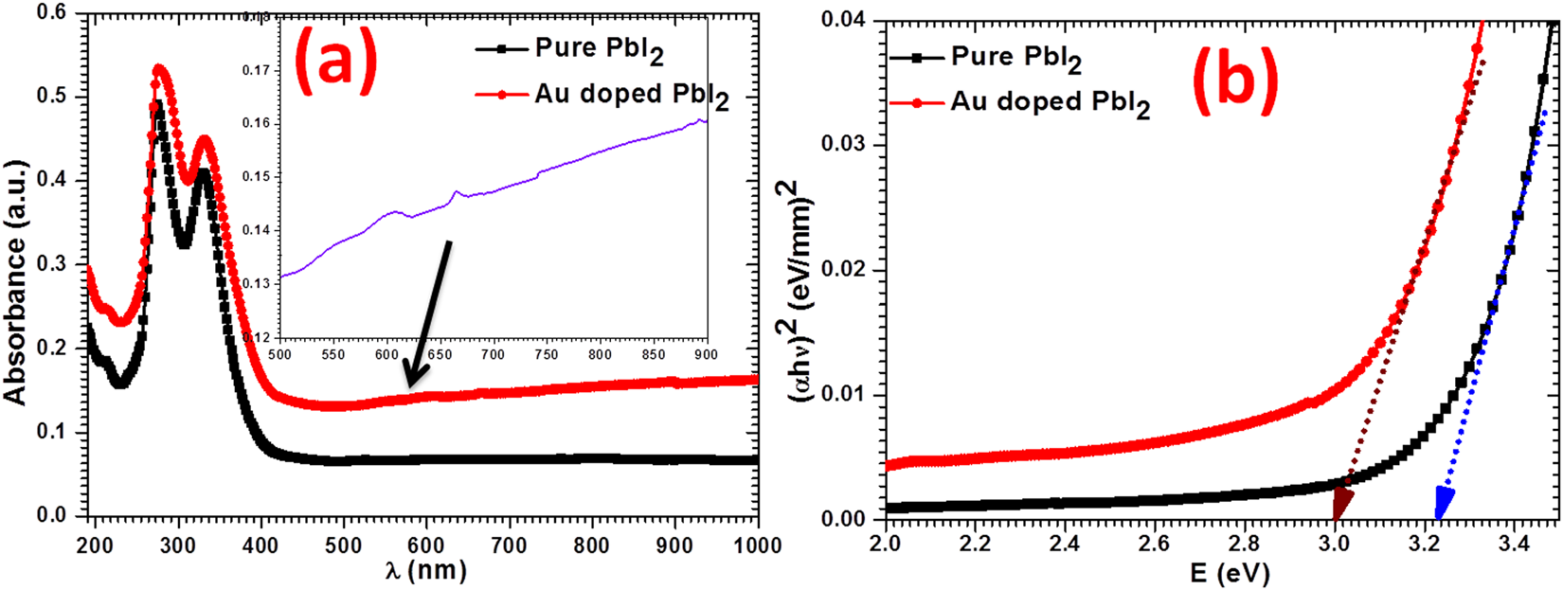
Examination of (**a**) absorbance and (**b**) energy gap for pure and Au doped PbI_2._

Figure 5(a) showed the measured absorbance spectra at room temperature. From the figure, it can be noticed that the Au doped PbI_2_ single crystalline nanosheets show more absorbance in the visible light region than the pure PbI_2_ nanorods with two absorption peaks between 230 nm to 420 nm. The enhancement in the absorption provides clear evidence on AuNPs formation on PbI_2_ SCNSs which may be seen in the last SEM image while watching it very closely. Moreover, Au doped PbI_2_ showed two absorption peaks at ~604 and ~665 nm in wavelength range of 500–900 nm (see inset of Fig. 5(a)); these peaks are maybe appeared due to absorption induced by surface plasmon resonance (SPR) that lies at ~630 nm wavelength and for Au nano colloid and Au doped films^51–54^. Absorbance for pure Au nanoparticles is reported at 525 nm^47^. It may be mentioned here that the absorbance spectra are recorded in DMF by making the transparent with a light yellow color solution of the prepared nanostructures. SPR is caused by collective oscillations of free electrons in metallic nanoparticles persuaded by exterior electromagnetic radiation, and its spectral features are strongly based on properties of mutual nanoparticles and host matrix^53^. A clear red shift in absorption edge is observed towards the higher wavelength in the nanosheets compared to nanorods. Two absorption bands are observed in the absorbance data which were used to determine the energy gap through Tauc’s plot achieved by Tauc’s formula and depicts in Fig. 5 (b). As the titled material is a well known direct band gap material so the value of energy gap ${E}_{g}$, is computed from graphs corresponding to joining point, where ${(\alpha h\nu )}^{2}=0$, of incidental straight line of the curve ${(\alpha h\nu )}^{2}$ vs. $h\nu$, at x-axis, where $h\nu$ and $\alpha$ are known as photon energy and absorption coefficient, respectively. Here, to evaluate the $\alpha$ values the Beer-Lambert relation is applied, i.e., $\alpha \,=\,2.303\,A/t$, where A is absorbance and t is the width of quartz cuvette (10 mm) used for the measurement. The calculated values of ${E}_{g}$ for pure and doped PbI_2_ are 3.23 eV and 3.00 eV, respectively. Decrease $({\rm{\Delta }}{E}_{g}=0.23\,eV)$ in the band gap of PbI_2_ is observed due to Au doping, however, band gap values are much larger than bulk value (i.e., 2.27 eV)^16^. These band gaps are noted to be analogous to recent literature values^55,56^. Such a reduction in band gap is also reporteda for Au doped TiO_2_ nanoparticles^51^. The higher value of band gap may indicate the occurrence of the confinement effect in prepared products. Such a high band gap value of prepared nanostructures makes them suitable for electro-optic devices^57–61^.

### Photoluminescence (PL) analysis

**Figure 6:**
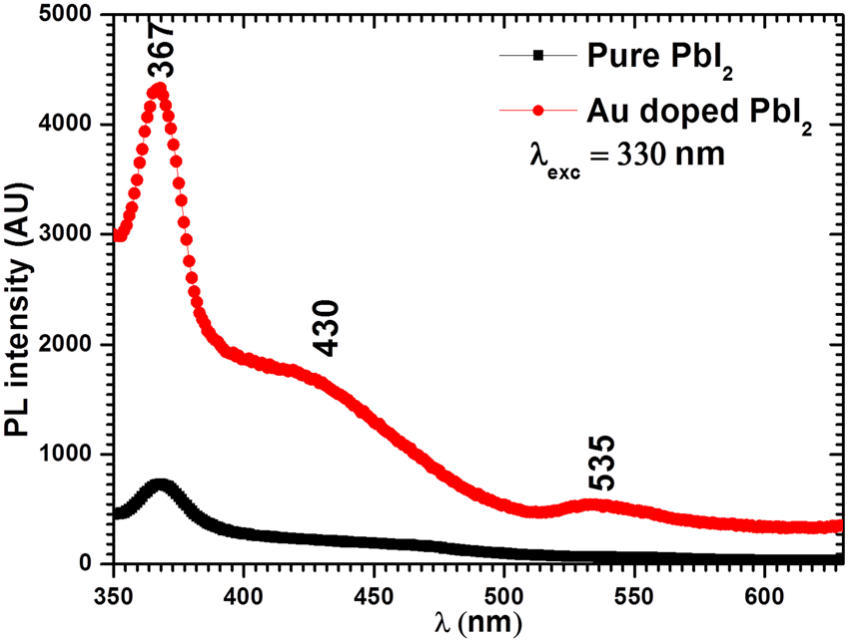
Photoluminescence spectra for pure and Au doped PbI_2_.

PL emission spectra recorded for both nanostructures at the excitation wavelength, ${\lambda }_{exc}=330\,nm$ are shown in Fig. 6. This spectra shows three distinct emission peaks at about 367 (3.380 eV), 430 (2.885 eV) and 535 nm (2.319 eV), however in spectrum of pure these emission peaks are observed at about 368 (3.371 eV) (UV-C or near violet emission) and 467 nm (2.656 eV) (blue emission) and 550 nm (2.256 eV) (green emission). The enhancement in intensity of PL emission is found by Au doping. The emission peaks at 2.656 eV and 2.256 eV are found to be shifted towards the higher energy by 0.229 eV and 0.063 eV in Au doped PbI_2_ nanosheets. The broad emission peak at 2.256 eV in pure and 2.319 eV in doped is assigned to G band that ascends from recombination of a trapped carrier that may be due to deep imperfection at interior or surface of the nanostructures. This emission peaks may also be assigned to D band which is associated to donar-acceptor pairs. Another broad emission peak at 2.656 eV in pure and 2.885 eV in doped PbI_2_ is may be attributed to bound excitons (E_B_), and peaks at 3.371 eV and 3.380 eV in pure and doped PbI_2_, respectivily, are occurred due to free excitons (E_F_)^55,62,63^. All the observed emissions are shifted towards higher energy or lower wavelength.

### Dielectric and ac electrical conductivity analyses

**Figure 7:**
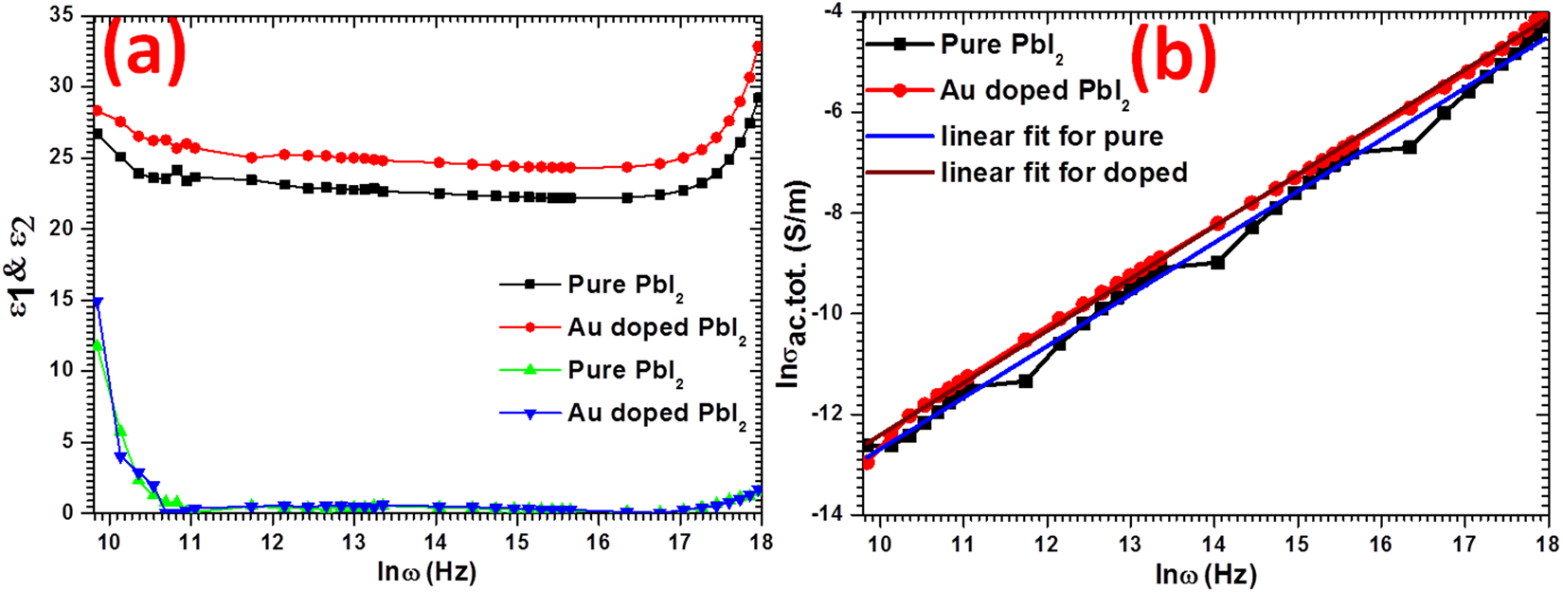
Examination for (**a**) *ε*_1_ & *ε*_2_ and (**b**) ln *σ*_*ac*.*tot*._ of pure and Au doped PbI_2._

Dielectric values of constant $({\varepsilon }_{1})$, loss (${\varepsilon }_{2}$) and total alternating current electrical conductivity $({\sigma }_{ac.tot.})$ were calculated by measuring the C, Z, and tanδ values of fabricated nanosheets in 3 kHz to 10 MH frequency range. Values of ${\varepsilon }_{1}$ and ${\varepsilon }_{2},$ were calculated using the equation:

$${\varepsilon }_{1}=\frac{\,C\times \,t\,}{{{\rm{\varepsilon }}}_{0}\times A\,}\,{\rm{and}}\,{\varepsilon }_{2}=tan\delta \times {\varepsilon }_{1},$$

where t is thickness, ${{\rm{\varepsilon }}}_{0}$ is vacuum space permittivity and A is area. The calculated values of ${\varepsilon }_{1}$ and ${\varepsilon }_{1}$ over a wide range of frequencies are depicted in Fig. 7(a). The stable values of ${\varepsilon }_{1}$ are obtained in the tested frequency region and that sanctions their application in optoelectronic devices. The ${\varepsilon }_{1}$ value is enriched for Au doped PbI_2_ (25) nanosheets compared to nanorods of pure PbI_2_ (22). The attained electrical values in current work are comparable with other reported work in the literature on nano and bulk PbI_2_^23,38,64^. As we have performed our studies over a high frequency range, the electric field dipole is not obeying the alternating field^65–69^. The ${\varepsilon }_{2}$ plot shows a same trend as of ${\varepsilon }_{1}$, the initial decrease in the value of ${\varepsilon }_{2}$ was observed, however at the higher frequency it becomes stable and very low which proves that the prepared nanosheets are low defective. Such nanosheets will be more effective in various applications.

Moreover, the ${\sigma }_{ac.tot.}$ values were calculated from the equation: ${\sigma }_{ac.tot.}=\frac{l}{Z\times A}$ and shown in Fig. 7(b) with frequency. The values of ${\sigma }_{ac.tot.}$ was enriched for Au doped PbI_2_ which indicates better electrical properties that can be more efficient in semiconductor devices. Also the enhancement in the values of ${\sigma }_{ac.tot.}$ was noticed with frequency which means it follows the universal frequency power law. The conduction behavior was analyzed using Jonscher law^70^, ${\sigma }_{ac.tot.}={\sigma }_{dc}+B{\omega }^{s}$, here, s is a frequency exponent and other symbols have usual meanings. The plot between $ln{\sigma }_{ac.tot.}$ and $ln\omega$ has been shown in Fig. 7(b) from which the value of s was obtained through the slope of this plot, and found below 1 for both samples. This means that the value of s is <1, which imply that the behavior of charge carriers is managed by correlated barrier hopping (CBH)^56^. Generally, for unordered systems, charge carriers progression is mainly deal with two physical actions known as CBH and quantum mechanical tunneling. Though, the exact character of charge transfer might be explicate with temperature disparity of s^56^.

### Electrical properties analysis

**Figure 8:**
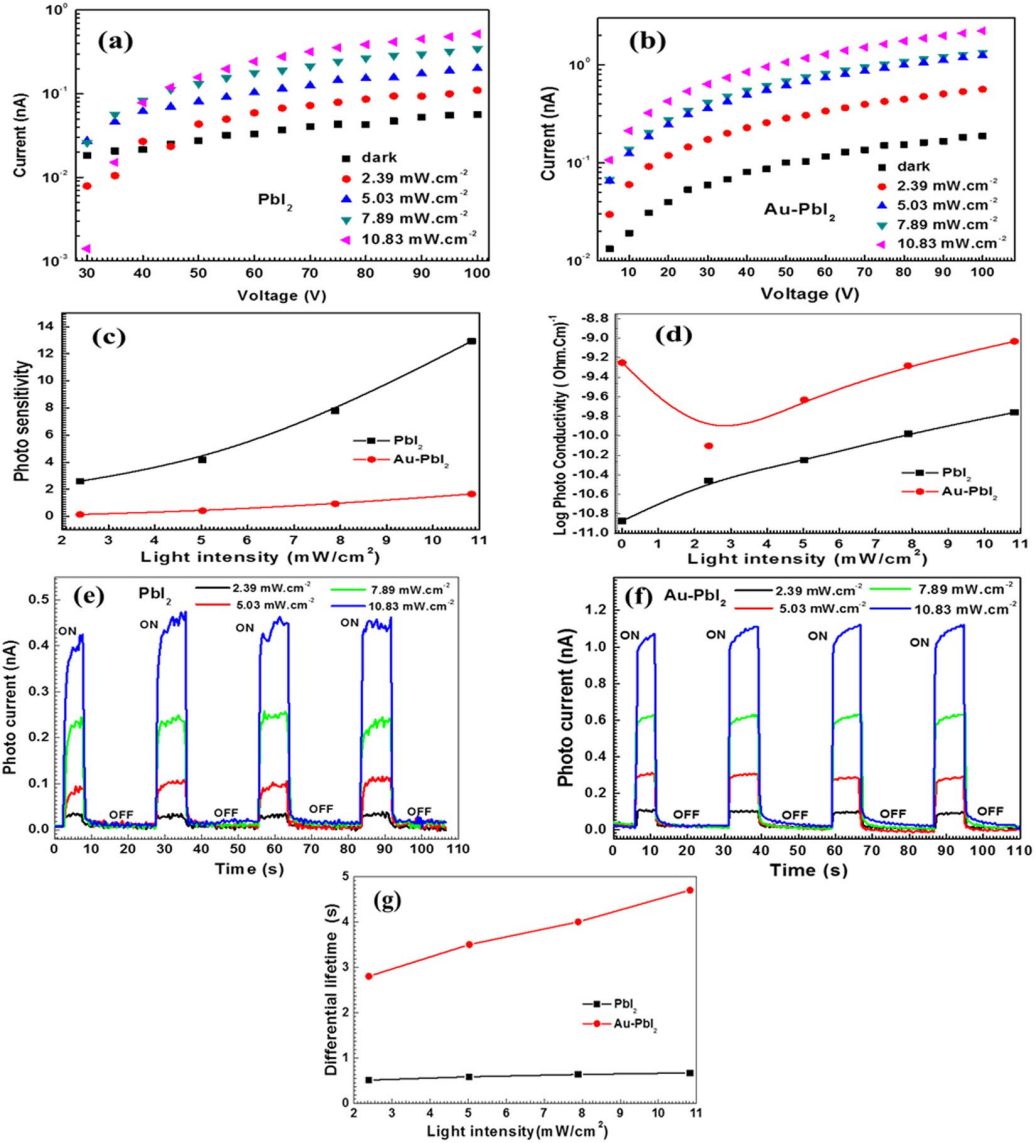
Current-voltage characteristics of (**a**) PbI_2_ and (**b**) Au-PbI_2_ at different incident light intensities, (**c**) Photosensitivity and (**d**) Photoconductivity as a function of light intensity, Time-resolved photoresponse of (**e**) PbI_2_ and (**f**) Au-PbI_2_ at different incident light intensities. (**g**) Differential lifetimes.

Lead iodide, Lead iodide based and other inorganic perovskites are reported to be good photodetectors^20,71–74^. Effect of Au doping and annealing on photoswitchability of ZnO is also reported previously^75,76^. Figure 8(a,b) shows I-V characteristics of PbI_2_ and Au-PbI_2_ at different incident light intensities along with the dark current reference. It is seen that the light current increases with incident light intensity. The (dark current) ON/OFF ratio values are (0.056 nA) 9 and (0.187 nA) 12 at 100 V for PbI_2_ and Au-PbI_2_, respectively. The light intensity power dependence of photoconductivity in Fig. 8(c) compares the photosensitivity of PbI_2_ and Au-PbI_2_ while Fig. 8(d) clearly shows photoconductivity enhancement in Au-PbI_2_ over PbI_2._ To investigate the stability of PbI_2_ nanosheet based photodetectors, the photo-response is measured by turning ON/OFF the light periodically at a constant interval for multiple cycles. In the OFF state of the laser, the photodetector has a low current (~0.01 nA) while the ON state increases the photodetector current rapidly to a saturation value. The low dark current indicates low noise and hence, higher device sensitivity. The switching mechanism can be easily understood in the previous report on Au doped ZnO by Mishra *et al*.^75^. The time-resolved photo-responses at various illumination powers are shown in Fig. 8(e,f). The photocurrents displayed no obvious deviations after 120 s, implying the good photodetector stability and reversibility. Also, the ON/OFF switching behavior retains well and is reversible. The differential lifetime [Fig. 8(g)] as a function of light intensity shows an enhancement of a factor of 6 in Au-PbI_2_ over PbI_2._ The response time and recovery time are found to be improved in Au-PbI_2_. From Fig. 8, it clear that Au doped PbI_2_ nanosheets exhibit better electrical properties than pure PbI_2._ The currently prepared nanosheets showed comparable electrical properties as reported for ultrathin as well as large PbI_2_ single crystal^19,20^.

## Conclusions

An efficient and rapid microwave-assisted synthesis of hexagonal single crystalline nanosheets of 1 wt% Au doped PbI_2_ has been described in this research. Structural characterization (XRD, SEM, TEM) revealed the homogenous distribution of polycrystalline Au nanoparticles (~40 nm) decorating PbI_2_ nanosheets. The vibrational modes are observed to be red-shifted compared to the bulk values of the studied material due to the confinement effects. While absorbance spectra displayed a new band at ~604 nm in doped nanostructures which may be due to the presence of Au. Optical energy gap was reduced from 3.23 eV to 3.00 eV when PbI_2_ was doped with Au nanoparticles. The intensity of PL emission was enhanced many folds in Au doped PbI_2_ due to the SPR of Au metal nanoparticle. This results in enhancing the excitation and emission rate of PbI_2_ nanosheets in the localized electromagnetic field. Dielectric constant value is enriched from 22 to 25, and total ac electrical conductivity was also found to be enhanced in Au doped samples. Au doped PbI_2_ nanosheets give a better photoconductive response, and the fabricated photodetector from single crystalline nanosheets show promising, stable, switchable photo-response. Such results give a cutting-edge idea in recognition of enhanced photoluminescent, photoconductor and electrical devices fabricated based on the Au-PbI_2_ single crystalline nanosheets.

## Electronic supplementary material

Supplementary data file
