# Supplementary material for: A facile synthesis of Au-nanoparticles decorated PbI2 single crystalline nanosheets for optoelectronic device applications
Source: Sci Rep. 2018 Sep 14;8:13806. doi: 10.1038/s41598-018-32038-5 (PMC6138657; doi:10.1038/s41598-018-32038-5)
Supplement: Supplementary file 1 — Supplementary data file [file 41598_2018_32038_MOESM1_ESM.pdf]

A facile synthesis of Au-nanoparticles decorated PbI_2 single crystalline nanosheets for optoelectronic device applications

Mohd. Shkir[1], I.S. Yahia[1], V. Ganesh[1], Y. Bitla[2], I.M. Ashraf[1,4], Ajeet Kaushik[3], S. AlFaify*[1]

[1]*Advanced Functional Materials and Optoelectronics Laboratory (AFMOL), Department of Physics, College of Science, King Khalid University, Abha 61413, P.O. Box 9004, Saudi Arabia*
[2]*Department of Physics, Indian Institute of Science, Bangalore 560012 India*
[3]*Centre of Personalized Nanomedicine, Institute of Neuroimmune Pharmacology, Department of Immunology, Herbert Wertheim College of Medicine, Florida International University, Miami, FL-33199 USA*
[4]*Department of Physics, Faculty of Science, Aswan University, Aswan , Egypt*

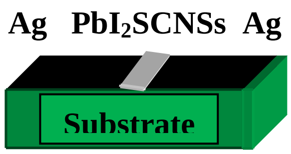

Scheme I: Simple design of sample for electrical measurements

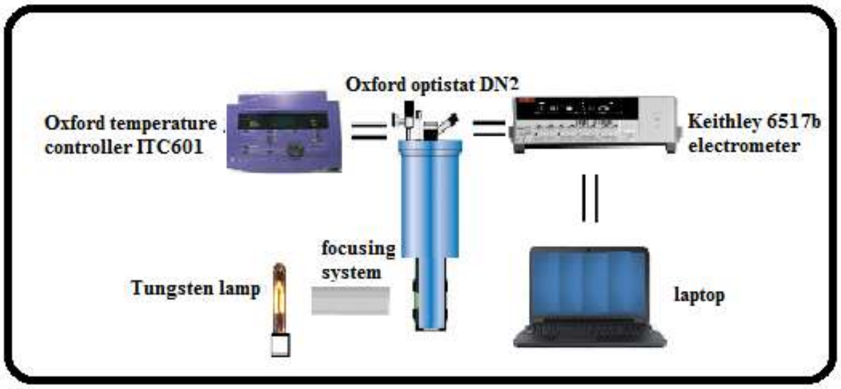

Scheme II: Schematic diagram for photoconductivity measurement
